# Supplementary material for: Adaptation of Dinoroseobacter shibae to oxidative stress and the specific role of RirA
Source: PLoS One. 2021 Mar 29;16(3):e0248865. doi: 10.1371/journal.pone.0248865 (PMC8007024; doi:10.1371/journal.pone.0248865)
Supplement: S1 Fig — Cells were grown aerobically in seawater medium. At an OD at 578 nm (OD578) of 0.5 (exponential growth phase), different concentrations of the oxidants were added to the cultures. H2O2: 0mM (•), 10 mM (■), 20 mM (▲), and 30 mM (▼). Diamide: 0 mM (•), 0.5 mM (■), 0.65 mM (▲), 0.8 mM (▼), and 1.0 mM (○) Paraquat: 0 μM (•), 10 μM (■), 15 μM (▲), 30 μM (▼), and 90 μM (○). For each concentration one representative growth curve is represented. (PDF) [file pone.0248865.s004.pdf]

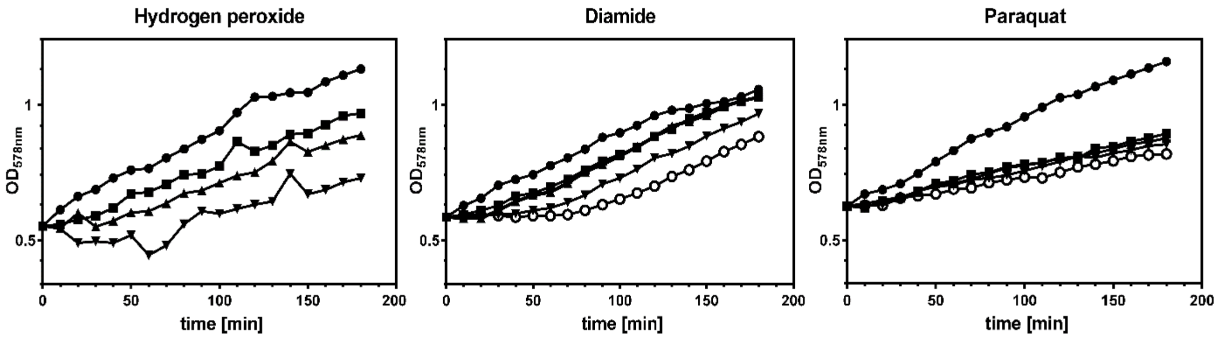

Figure S1 Effect of oxidative stress on growth of *D. shibae*. Cells were grown aerobically in seawater medium. At an OD at 578 nm (OD_{578}) of 0.5 (exponential growth phase), different concentrations of the oxidants were added to the cultures. H_2O_2: 0mM (•), 10 mM (■), 20 mM (▲), and 30 mM (▼). Diamide: 0 mM (•), 0.5 mM (■), 0.65 mM (▲), 0.8 mM (▼), and 1.0 mM (○) Paraquat: 0 µM (•), 10 µM (■), 15 µM (▲), 30 µM (▼), and 90 µM (○). For each concentration one representative growth curve is represented.
