# Supplementary material for: Adaptation of Dinoroseobacter shibae to oxidative stress and the specific role of RirA
Source: PLoS One. 2021 Mar 29;16(3):e0248865. doi: 10.1371/journal.pone.0248865 (PMC8007024; doi:10.1371/journal.pone.0248865)
Supplement: S1 File — (PDF) [file pone.0248865.s005.pdf]

Supporting Material and Methods

Adaptation of *Dinoroseobacter shibae* to oxidative stress and the specific role of RirA

Nicole Beier[1,2], Martin Kucklick[1,2], Stephan Fuchs[3], Ayten Mustafayeva[1,2], Maren Behringer[1], Elisabeth Härtig[1], Dieter Jahn[1,4], and Susanne Engelmann[1,2,4]

[1]Technische Universität Braunschweig, Institute for Microbiology, Germany

[2]Helmholtzzentrum für Infektionsforschung Braunschweig, Microbial Proteomics, Germany

[3]Robert-Koch-Institut, Berlin, Germany

[4]Technische Universität Braunschweig, Braunschweig Integrated Centre of Systems Biology (BRICS), Germany

Running title: Oxidative stress response in *D. shibae*

Construction of *a* ∆*rirA* deletion mutant for *Dinoroseobacter shibae*

To delete the *rirA* gene (Dshi_1660) in *D. shibae* DFL-12, up- and downstream DNA sequences of *rirA* were fused with a gentamicin resistance DNA cassette and inserted into the genome *via* double homologous recombination events. For amplification of the upstream (1289 bp) and downstream DNA fragment (1813 bp) of *rirA*, chromosomal DNA of *D. shibae* DFL-12 and primer pairs EH611/612 and EH615/616 were used. The DNA fragment coding for the gentamicin resistance cassette (872 bp) was amplified using plasmid DNA of pBBR1-MCS-5 [1] and oligonucleotides EH613 and EH614. The three DNA fragments were annealed and the resulting 3948 bp DNA fragment was amplified using primer pair EH611/616. After cleavage with *Hin*dIII and *Kpn*I, the DNA fragment was cloned into the suicide vector pEX18Tc [2], which was cleaved with the same enzymes. The resulting plasmid pEX18Tc∆*rirA* was transformed into the *Escherichia coli* ST18 donor strain [3] and subsequently transferred into *D. shibae* wild type strain DFL12^{T} by biparental mating. Gentamicin resistant strains were selected using half-concentrated marine agar plates containing 80 µg/ml gentamicin [4]. Replacement of *rirA* by the gentamycin resistance cassette was verified *via* multiplex PCR. Here, the primer pair EH590/599 generating a 490 bp DNA fragment containing the upstream region of *rirA* and the gentamicin resistant cassette was used as a positive control and EH590/591 generating a 762 bp DNA fragment, covering the native *rirA* gene with its upstream region, as negative control.

Table 1
Oligonucleotides used in this study

Name	Sequence (5´to 3´)[a]
EH590	GATCGGCTTGATACGTTCCT
EH591	GATGACTTGTCCGAGATGGT
EH599	GATCGGCTTGATACGTTCCT
EH611	GCG*AAGCTT*AGTTCGCGGGCAGTATCA (*Hin*dIII)
EH612	GCGGTGTTACGGTCTGCT*CTGCAGT*ACGT (*Pst*I)
EH613	TGCA*CTGCAG*GGCGTTGTGACAATTTAC (*Pst*I)
EH614	CGTTTCCACGGTGTGCGTC*GGATCC*CAT (*Bam*HI)
EH615	ATG*GGATCC*ATCGACGGAGAAACATTGATG (*Bam*HI)
EH616	TGACGAGAAGACCCGTACGC*GGTACC*CC (*Kpn*I)

[a] restriction sites are given in italic letters

Construction of a ∆*rirA* complementation mutant of *D. shibae*

To complement the loss of RirA in the ∆*rirA* deletion mutant, the *rirA* gene fused with StrepII tag DNA at the N-terminal site of RirA was amplified using plasmid DNA of pET52b*rirA* and the oligonucleotides EH641 and EH642 containing an *Nde*I and a *Pst*I restriction sites, respectively. The obtained DNA fragment was inserted into the multiple cloning site of pRhokS [5] and in this way fused with the constitutive promoter PaphII. The resulting vector pRhokS*rirA* was used for complementation. To this end the vector was transferred into the ∆*rirA* deletion mutant DS010 *via* biparental mating using *E. coli* ST18 as donor strain. Selection of plasmid

containing strains was performed on half-concentrated marine agar plates with 15 µg/ml chloramphenicol.

LC-MS/MS-Analyses

Aliquotes of 20 µg protein crude extracts in loading buffer (3.75% (V/V) glycerol, 1.25% (V/V) ß-mercaptoethanol, 0.6% (w/v) SDS, 0.0014% (w/v) bromophenol blue16.5 mM Tris, pH6.8) were separated via one-dimensional SDS polyacrylamide gel electrophoresis (15 mA per gel) according to Laemmli [6] with the following modifications: for the separation gel: 12% (w/v) acrylamide gel (with 0.32% bisacrylamide), 0.375 M Tris-HCL (pH 8.8), 0.255% (w/v) SDS, 0.062% (w/v) APS, and 0.062% (v/v) TEMED; for the stacking gel: 5% (w/v) acrylamide (with 0.13% (w/v) bisacrylamide), 0.125 M Tris-HCl (pH 6.8) 0.25% (w/v) SDS, 0.075% (w/v) APS, and 0.075% (v/v) TEMED. In gel digestion of proteins was carried out as described previously [7] by dividing each lane into eight subsamples with similar protein amounts which were densitometrically determined using AIDA image analysis software (version 4.15., Raytest Isotopenmeßgeräte GmbH, Straubenhardt, Germany) and using a digestion buffer containing 50 mM Tris/HCl (pH 7.6) and 1 mM $CaCl_2$. Extraction and desalting of the resulting peptides were done according to Lassek and coworkers [8] starting with an additional extraction step using acetonitrile.
For liquid chromatography – coupled tandem mass spectrometry (LC-MS/MS) analyses a nanoAQUITY Ultra Performance Liquid Chromatography System (Waters Corporation, Milford, MA, USA) was connected to an LTQ Orbitrap Velos Pro mass spectrometer (Thermo Fisher Scientific Inc). Peptides from each gel piece were solved in 3 % acetonitrile and 0.1 % formic acid, centrifuged for 20 min at 109,000 x g and the supernatant was loaded onto a BEH C18 column, 130 Å, 1.7 µm, 75 µm x 250 mm at a flow rate of 0.35 µl min^{-1} (Waters Corporation). Elution of peptides from the column was performed using a 222 min gradient starting with 3.7% buffer B (80% acetonitrile and 0.1% formic acid) and 96.3% buffer A (0.1% formic acid in Ultra-LC-MS-water): 0 to 30 min 3.7% B; 30 to 65 min 3.7 to 22.1% B; 65 to 70 min 22.1 to 23.9% B; 70 to 97 min 23.9 to 29.3% B; 97 to 134 min 29.3 to 37.8% B; 134 to 167 min 37.8 to 48.3% B; 167 to 194 min 48.3 to 62.5% B; 194 to 211 min 62.5 to 99% B; 211 to 213 min 99% B; 213 to 218 min 99 to 3.7% B, 218 to 222 min 3.7% B.

Primary MS scans were performed in the Fourier transformation mode scanning an m/z of 400-2000 with a resolution (full width at half maximum at m/z 400) of 60,000 and a lock mass of 445.12003. Primary ions were fragmented in a data-dependent collision induced dissociation mode. The 20 most abundant precursor ions were analysed by the LTQ ion trap with an exclusion time of 13 s. The following ionization parameters were applied: normalized collision

energy: 35, activation Q: 0.25, activation time: 10 ms, isolation width: 2 m/z, charge state: ≥ +2. The signal to noise threshold was set to 2000.

MS/MS Data analyses

MS/MS raw files were analysed using MaxQuant (Max Planck Institute of Biochemistry, Martinsried, Germany, www.maxquant.org, version 1.5.2.8) and the following parameters: peptide mass tolerance: 5 ppm; tolerance for fragment ions: 0.6 Da; variable modification: methionine oxidation, fixed modification: carbamidomethylation; a maximum of three modifications per peptide was allowed; the fixed false discovery rate (FDR) was set to 1% for peptides and proteins. All samples were searched against a database containing all protein sequences of *D. shibae* DLF 12 extracted from NCBI at 05/09/16 with a decoy mode of reverted sequences and common contaminants supplied by MaxQuant. MS data filtering and statistical analyses were done using the proteinGroups.txt output file of MaxQuant. For reliable identifications only proteins with a minimum of two unique peptides each of them detected by at least one MS/MS scan in two different samples were considered. For protein quantification, a labelfree quantification method using LFQ intensities was applied [9]. If the sum of raw intensities for three replicates of an identified protein was ≤ 500.000, LFQ intensities were set to zero.

A Post hoc analysis was performed with the Perseus software (Version 1.5.0.15, www.maxquant.org). For statistical analyses LFQ intensities were transformed into log2 values and standardized using Z-score. To evaluate whether the amount of a given protein changed in response to stress, a p-value based ANOVA (p-value ≤ 0.05) was applied. Significantly changed expression profiles were subsequently tested using Student's t-test (p-value ≤ 0.05) to compare the standardized LFQ intensities of stressed samples of each time point with those of control samples (t_0). Finally, only proteins whose amount changed by at least 1.5 fold were considered as significantly differently expressed. When signal intensities were below the detection level either in control or in stressed samples no induction ratios have been calculated. Relevant proteins, which were identified in at least two replicates and showed mean LFQ intensities above the standard error, were termed as "down" (solely present in control samples) and "up" (solely present in stressed samples) proteins and added to the list of significantly changed proteins.

The mass spectrometry proteomic data have been deposited in the ProteomeXchange Consortium via the PRIDE partner repository [10] with the dataset identifier PXD013791.

Subcellular localization of identified proteins was predicted using LocateP v2 (http://bamics2.cmbi.ru.nl/websoftware/locatep2/locatep2_start.php). Replicon coding information was taken from Rosy v2 (http://rosy.tu-bs.de/index.php). Protein products, gene

names and assignment to functional categories or metabolic pathways were extracted from UniProt (https://www.uniprot.org/) and intergrated microbial genomes database (https://img.jgi.doe.gov/).

References

[1] M. E. Kovach, P. H. Elzer, D. S. Hill, G. T. Robertson, *et al.*, *Gene* **1995**, 166, 175.
[2] T. T. Hoang, R. R. Karkhoff-Schweizer, A. J. Kutchma, H. P. Schweizer, *Gene* **1998**, 212, 77.
[3] S. Thoma, M. Schobert, *FEMS Microbiol. Lett.* **2009**, 294, 127.
[4] T. Piekarski, I. Buchholz, T. Drepper, M. Schobert, *et al.*, BMC Microbiol. **2009**, 9, 265.
[5] N. Katzke, S. Arvani, R. Bergmann, F. Circolone, *et al.*, Protein Expr. Purif. **2010**, 69, 137.
[6] U. K. Laemmli, Nature **1970**, 227, 680.
[7] M. Toyofuku, B. Roschitzki, K. Riedel, L. Eberl, J. Proteome Res. **2012**, 11, 4906.
[8] C. Lassek, M. Burghartz, D. Chaves-Moreno, A. Otto, *et al.*, Mol. Cell. Proteomics **2015**, 14, 989.
[9] J. Cox, M. Y. Hein, C. A. Luber, I. Paron, *et al.*, Mol. Cell. Proteomics **2014**, 13, 2513.
[10] J. A. Vizcaino, A. Csordas, N. del-Toro, J. A. Dianes, *et al.*, Nucleic Acids Res. **2016**, 44, D447.
