# Supplementary material for: Adaptation of Dinoroseobacter shibae to oxidative stress and the specific role of RirA
Source: PLoS One. 2021 Mar 29;16(3):e0248865. doi: 10.1371/journal.pone.0248865 (PMC8007024; doi:10.1371/journal.pone.0248865)
Supplement: Attachment — Submitted filename: Response to the reviewers.docx [file pone.0248865.s006.docx]

Dear Reviewers,

We are very grateful for your comments and suggestions that were very helpful to improve our manuscript significantly. As recommended, we rigorously revised the RirA part of the manuscript to present more clearly the role of RirA for adaptation to peroxide stress. To this end, two novel figures were included in the manuscript: Figure 5 and Figure 7. In Figure 5, expression profiles of the iron-responsive regulators RirA, Irr and IscR as well as the three OxyR homologues in response to the different oxidants are shown. Figure 7 represents growth curves of the wild type as well as of the *rirA* deletion and the *rirA*-complemented strain under control conditions and in response to peroxide stress and support the data presented in Table 1. Moreover, Figure 3 and Figure 4 (formerly Figure 2B) were changed according to suggestions of Reviewer #1 and now additionally present protein expression data obtained for the *rirA* mutant in response to peroxide stress. We agree with Reviewer #2 that the clustering of *rirA* dependent proteins was somewhat confusing and very preliminary and deleted this part from the manuscript. Now we are focusing on proteins upregulated in the *rirA* mutant. It is very likely that among them are those playing a role in adaptation to peroxide stress. Moreover, we are discussing the role of iron-regulated proteins in oxidative stress resistance. Their peroxide responsive induction is missing in the absence of RirA. Hence, the increased sensitivity of wild type cells to peroxide stress may also be explained by disorders of the cellular iron homeostasis triggered by the induction of iron-regulated proteins.

In the following, we specifically respond to the comments given by the two reviewer:

**Reviewer #1:**

This manuscript presents a highly detailed analysis of proteomic changes in the marine bacterium Dinoroseobacter shibae in response to oxidative stress. The authors analyze temporal changes in the bacterial proteome in response to three different types of oxidative stress. In addition, they also examine a mutant lacking the iron-responsive transcriptional repressor RirA. An impressive amount of work has gone into compiling the results presented.
However, the manuscript is rather confusing as written and could benefit from changes in how the data is presented. As stated in the abstract, the authors seek to demonstrate that “RirA dependent proteins are important for oxidative stress resistance”. However, the link of the RirA regulator to the changes in response to oxidative stress (peroxide stress in particular) is lost in the manuscript writing and should be better highlighted.

My major comments are listed below:
1. The authors introduce RirA as a likely iron-responsive regulator (lines 263-264 and 387-388) and seek to address its role in oxidative resistance in D. shibae. Furthermore, in lines 443-444, the authors state that “The most striking response of D. shibae to oxidative stress was the induction of proteins involved in iron metabolism.” It is therefore puzzling to me that the authors choose to highlight not iron-related proteins, but a putative glutathione S-transferase, ClpB and Hsp20 as particularly interesting candidates identified with the mutant in the abstract (lines 40-45). It is also not made clear why they are particularly interesting; I think it would be a stronger paper if the focus in the abstract remains in the context of iron- related RirA mechanisms.

**Response**: According to the reviewer´s suggestions, we rigorously revised the RirA part of the manuscript. We now provide an explanation why we think that the above-mentioned proteins might play a role for oxidative stress resistance. Moreover, we speculate about the effects proteins that are involved in iron metabolism and induced in peroxide stressed cells may have on cell viability.

Line 40: Adding the term “iron-responsive” in front of the term “regulator RirA” may be more informative for the reader.

**Response**: Has been added as suggested.

2. Fig. 1A shows that the growth rate is affected for maybe 30 minutes after exposure to 10 mM hydrogen peroxide, but then largely recovers subsequently. Why is that not reflected in the recovery of ATP levels at later times after exposure to 10 mM hydrogen peroxide (Fig. 3)?

**Response:** After exposure to hydrogen peroxide, the ATP level dropped to 60% within 60 min and slightly increased thereafter. Since the growth of D. *shibae* already recovered 30 min after exposure to hydrogen peroxide, we consider that the drop of ATP might not be the main reason for growth inhibition in *D. shibae* under these conditions.

3. With reference to the rirA deletion mutant, it would be helpful and important to provide some information regarding its phenotype. Does it grow like the wild type, and to the same density? Perhaps a growth curve relative to wildtype could be presented in a figure along with the Table 1 details.

**Response**: Under control conditions, we did not observe a different growth phenotype for the *rirA* mutant compared to the wild type. This information is now presented in the manuscript (lines 435 - 436). Moreover, we added a new figure to the manuscript (Figure 7) demonstrating more clearly the growth phenotype of the wild type, the *rirA* mutant and the *rirA* complemented mutant in response to hydrogen peroxide and under control conditions.

4. The results with the mutant are difficult to grasp as written (lines 401- 459), and I am afraid the impact would be lost on the reader. The authors should make an effort to integrate these results with those of the wild type in a clearer way.

**Response:** The chapter describing the effects of RirA on protein expression in *D. shibae* before and after exposure to hydrogen peroxide was rigorously revised.

Fig. 2B very nicely displays changes in specific proteins over time in response to oxidative stress (although I had trouble reading the font clearly). I would suggest including in Fig. 2B corresponding changes in the mutant – this will make clear to the reader at a glance which of the stress-responsive proteins are dependent on RirA. At the very least, this should be done with regard to iron metabolism (lines 443-449).

**Response:** Figure 3 and Figure 4 (formerly Figure 2B) now additionally present protein expression profiles under peroxide stress conditions in a *rirA* mutant. Moreover, relative amounts of these proteins in the *rirA* mutant compared to the wild type are shown.

Additional points:
i. Line 97: “Rodeobacter”- a typo?

**Response:** was corrected

ii. Lines 144-145: The sentence was not clear to me: do the two samples indicate two out of three biological replicates?

**Response**: For identification of a peptide in a proteomics experiment, we need at least two MS/MS scans matching the theoretical MS/MS spectrum of this peptide. The two MS/MS scans have to be obtained from MS/MS analyses of two different samples or replicates belonging to the respective proteomics project. The sentence was modified in the manuscript (lines 147 – 149).

iii. Lines 167-169: the sentence does not read well?

**Response**: The sentence was modified (lines 170 - 172).

iv. Lines 169-170: it would be useful to state here the type of oxidative stress induced by each compound (peroxide, thiol etc.) for the benefit of the reader.

**Response**: This information was added as suggested.

v. Line 199. Please state the statistical test used.

**Response**: We used one way ANOVA. This information was added as suggested.

vi. Line 210: is it 63 proteins, not 73 that are changed with two or three of the oxidants?

**Response**: Thank you very much. We corrected this mistake.

vii. Line 231, one “to” needs to be deleted.

**Response:** was deleted

viii. Fig. 2B. Text is not clear. Please explain color code in the legend.

**Response**: The color code is now explained in the legend as well as in the figure (now Figs 3 and 4).

ix. Lines 263-264. It may be nice to have the data for these regulators presented in a figure. Perhaps it is included in the Iron metabolism section of Fig. 2B, but it is not legible.

**Response:** Expression profiles of the iron-responsive regulators RirA, IscR and Irr in response to the different oxidants are presented in Figure 5.

x. Lines 270-272. Since a reference to OxyR is made, please comment on presence or expression of an OxyR homolog in D. shibae.

**Response**: There are three OxyR homologues in *D. shibae*. We inserted a new Figure (Fig. 5) into the manuscript showing the expression profiles for these OxyR homologues in response to the different oxidants. Moreover, we provided additional information on amino acid conservation of the three OxyR homologues (lines 412 - 423).

**Reviewer #2:**

This paper examines the oxidative stress response of Dinoroseobacter shibae by looking at protein expression upon exposure to hydrogen peroxide, paraquat and diamide. Identified proteins included those involved in oxidative stress defense as well as a number of iron-regulated proteins, including RirA. The paper then makes an effort to identify RirA-regulated proteins involved in oxidative stress defense.

General issues - use of language. Aside from comma use errors, some run-on sentences, and awkward phrasing, there are a number of areas where the word choice is incorrect. The document should be close reviewed for grammar and clarity.

**Response**: We apologize for the mistakes we made. The revised manuscript was carefully checked regarding grammar, word choice and clarity.

A few selected examples
line 110 - “exposition to stress” should be “exposure to”

**Response**: was corrected

line 128 -“Peptides from each gel piece were solved in..” should be “purified using”

**Response**: was corrected

line 142 – “labelfree” should be “label-free”

**Response**: was corrected

line 144 - “A protein was considerably” should be “considered”

**Response**: was corrected

Line 157 - “exposition” again

**Response**: was corrected

Line 167-168 - “ sole carbon source in the dark, different” should be “…in the dark. Different…”

**Response**: was corrected

Line 180-181 - “in the presence of diamide of unstressed cells” – needs to be clarified

**Response**: “of unstressed cells” was deleted

Line 214 – “different ways in D. shibae, this was…” should be “…in D. shibae. This was..”

**Response**: was corrected as suggested

Line 217 – ferritine should be ferritin

**Response**: was corrected

Line 324-325 “ and gluconeogenesis has been shown to be a subject” should be “and gluconeogenesis have been shown to be subjects”

**Response**: was corrected

Figure 2b – Iron metabolism. The first item is “Soul heme-binding protein”. I suspect you meant “sole”

**Response:** The term “Soul” is correct.

Experimental – The protein expression data is all represented reasonably well in the figures and accompanying text, as is the analysis of ATP levels. I am not confident at all in the data presented for the RirA mutant in response to peroxide stress (Tab. 1 line 430-435). To begin with, a table is not the best way to present these data, particularly when you are looking at ranges of time (0-30, 30-120 min). A line graph would be best here.

**Response**: To support the data presented in Table 1, we inserted a novel Figure (Figure 7) representing growth curves of the wild type, the *rirA* mutant and the *rirA* complemented mutant under control conditions and in response to peroxide stress.

Additionally, there are some points in this figure that call it’s accuracy into question. Notably, the wild-type and complemented mutant showed negative optical density at the 0-30 minute time point. Also, none of the control strains showed any significant growth from the 0-120 minutes, but all samples showed increased growth in the same time period when exposed to peroxide. Increased survival might be a likely outcome for the rirA mutant, based on what you are proposing, but it is highly unlikely that oxidative stress is promoting growth for all strains involved.

**Response:** In Table 1, growth rates representing the number of cell divisions per hour for the respective strains during the indicated time intervals are shown. Negative growth rates obtained for the wild type and for the *rirA* complemented strain immediately after exposure to 10 mM H_2_O_2_ (0 – 30 min) indicate cell lysis. Subsequently, wild type and *rirA* mutant cells started to grow with nearly the same growth rates as the control cultures. In contrast, control cultures grew with a constant growth rate indicating that they are at logarithmic growth phase in the considered period. The data presented in Table 1 are now supported by Figure 7 showing the growth curves for the different strains under control conditions and after exposure to 10 mM H_2_O_2_.

For Figure 4, the way the data are presented does not make it clear what you are trying to show. Rather than just accession number examples of several representatives from each cluster, I would suggest a more in-depth analysis of what your representative proteins are predicted to do, how they are differentially regulated in the rirA mutant and why that change in regulation is associated with the observed rirA mutant phenotype. This figure just feels like a collection of information that has not been sufficiently analyzed for meaning.

**Response**: As already mentioned above, the clustering of *rirA* dependent proteins was deleted from the manuscript. To present the data for the *rirA* mutant more clearly, we focused on proteins upregulated in the *rirA* mutant and might thus playing a role in adaptation to peroxide stress. These are in particular those proteins involved in repair and degradation of oxidized cell structures such as DNA and proteins. Moreover, we discussed the role of iron-regulated proteins in oxidative stress resistance in *D. shibae*.
